# Rickettsial infection among military personnel deployed in Northern Sri Lanka

**DOI:** 10.1186/s12879-014-0688-8

**Published:** 2014-12-20

**Authors:** Ranjan Premaratna, Nimalka Ariyaratna, Champaka Attanayake, Wijesinghe Bandara, Nilmini Chandrasena, H Janaka de Silva

**Affiliations:** Departments of Medicine, Faculty of Medicine, University of Kelaniya, Ragama, Sri Lanka; Military Hospital, Colombo, Sri Lanka; Military Hospital, Palali, Sri Lanka; Department of Microbiology, Faculty of Medicine, University of Kelaniya, Ragama, Sri Lanka; Department of Parasitology, Faculty of Medicine, University of Kelaniya, Ragama, Sri Lanka

**Keywords:** Rickattsial infections, Military, Sri Lanka

## Abstract

**Background:**

Military personnel deployed in field actvities report on frequent tick bites. Therefore they may run the risk of exposure to rickettsial organisms.

**Methods:**

In order to assess the risk of exposure to rickettsial organisms, two groups of military personnel who were deployed in field activities of Nothern Sri Lanka were investigated. The first group was studied in order to assess the sero-prevalence of rickettsioses and consisted of soldiers who were admitted following injuries during field activities. The second group was studied to identify the incidence of acute rickettsioses during their acute febrile presentations. They were tested with IFA-IgG against spotted fever group rickettsioses (SFG), scrub typhus (ST) and murine typhus.

**Results:**

In the first group, 48/57 (84%) military personnel had serological evidence of exposure to rickettsioses (in all, IFA-IgG titer ≥ 1:128): 33/50 (66%) to SFG rickettsioses, 1/50 (2%) to ST and 14/50 (28%) had mixed titers for both (in all, titers were higher for SFG). While all of them were in military uniform most of the time and frequently slept on scrub land, 35/57 (61.4%) had never used insect repellents and none were on doxycycline prophylaxis. 48/57 (84%) had experienced tick bites during field activity.

In the second group, there were 49 who presented with acute febrile illness with a mean duration of 8.5 days (SD 3.2). 33/49 (67.3%) were serologically positive for acute rickettsioses (IgG ≥1:256); 26 (79%) due to ST and 7 (21%) due to SFG rickettsioses,

**Conclusions:**

Exposure to rickettsial disease was common among soldiers who were deployed in Northern Sri Lanka. Scrub typhus was the predominent species accounting for acute febrile illness. Further studies are needed to understand the reasons for very high sero-prevalence for SFG rickettsioses with no anticedent febrile illness. Use of preventive measures was not satisfactory. The high sero-prevelence of SFG rickettsioses is likely to interfere with serological diagnosis of acute SFG rickettsioses in this population.

## Background

Rickettsial infections are re-emerging in Sri Lanka [1],[2]. The history of rickettsioses dates back to the world wars when the troops arriving in Sri Lanka, then Ceylon, were attacked by chigger mites resulting in severe scrub typhus infection. The diagnosis of the illness had been based on the presence of eschars and high OXK titres [3]. Among the military most scrub typhus patients had presented with febrile illness. However some patients have had serious complications such as encephalitis, pneumonitis and deafness [3]. Recent literature from the country document re-emergence of spotted fever group rickettsioses (SFG), typhus fever and scrub typhus (ST) [1],[2],[4]. These infections are prevalent in almost all the parts of the island, however spotted fever group rickettsioses seems to predominate in the hilly central region and scrub typhus in the dry and wet lands of low country [1],[2],[4]. Although most SFG infections are sporadic and occur throughout the year, scrub typhus seems to have a seasonal occurrence [2].

The main vectors for scrub typhus (caused by *O. tsutsugamushi)* are chigger mites, and for SFG rickettsioses are tick species. Military personnel who are actively engaged in field activities complain of frequent tick bites and therefore may run the risk of developing rickettsial infections. We present the outcome of two study groups that were done in 2008 in order to understand the occurrence of rickettsioses among military personnel who were deployed in Northern Sri Lanka.

## Methods

### Group one

For the group one military personnel who were admitted to the Colombo North Teaching Hospital, Ragama, for management of injuries during field activities in early 2008 were recruited. The sero-prevalence rate for rickettsioses in the country is largely unknown however 56.3% mean sero-prevalence has been documented for four geographical regions [5]. As military includes individuals from many geographical regions within the country, their back ground sero-prevalence may vary. However since their activities expose them for insect and tick bites, we presumed that they may have had a higher risk for rickettsioses than the general population. We recruited a convenient sample for this preliminary study. After obtaining informed consent, they were interviewed by three experienced medical officers using a pre tested questionnaire. The questionnaire included data on demography, their service period in the area, activities they were engaged in, leasure activities, clothing, exposure to insects and ticks and its frequency, use of insect repellents and the frequency and history of febrile illness during the service period.

They were tested for serological evidence of rickettsioses using 1 ml serum sample collected for other routine investigations.

### Group two

The group two consisted of 49 consecutive admissions to Palali Military Hospital, Northern Sri Lanka, who developed an acute febrile illness during field activities. The study was carried out over a period of four months commencing from August 2008. They were tested for rickettsioses as part of investigation for febrile illness.

Serum samples were transported in ice to the Rickettsial Disease Diagnostic and Research Laboratory, Faculty of Medicine, University of Kelaniya for rickettsial antibody testing using Immuno Fluorescent Antibody (IFA) test against SFG rickettsioses and ST, using *R. conorii, R. typhi* and *O tsutsugamushi* antigens. In the first study, exposure to a rickettsial agent was defined when they had a IFA-IgG titre ≥1:64. In the second study the presumptive diagnosis of acute rickettsioses was based on the presence of acute febrile illness that is compatible with acute rickettsioses together with rapid deferversence with antirickettsial antibiotics and an IgG titre of ≥1:256 [6] and when they were negative for other causative aetiology for acute febrile illness [based on blood picture for malaria, blood culture for bacterial growth, serology for leptospirosis and typhoid].

### Ethical statement

Ethical approval for both studies were obtained from the Ethics Review committee of the Faculty of Medicine, University of Kelaniya, Sri Lanka. Informed written consent was obtained from all participants of group one and informed verbal consent was obtained from the participants of group two in order to use demographic, clinical and serological data without reveling their personal identity.

## Results and discussion

### Group one

The group one included 57 military personnel (all males) with a mean age of 25.8 (SD 5.5) years and a mean period of 6.7 (SD 5) years of active service in operational areas. They were from 20 out of 25 districts of Sri Lanka, however all 57 had served in the Northern Province and 13 had served in the Eastern Province of Sri Lanka in addition. 24/57 (42%) had a history of febrile illness during their service period (four were confirmed as malaria and the rest were undiagnosed). Although all of them were in military uniform most of the time, they had frequently slept on scrub land. 35/57 (61.4%) had never used insect repellents and the rest had used them infrequently. None of them were on doxycycline prophylaxis. 48/57 (84%) had experienced tick bites during field activities. 48/57 (84%) had serological evidence of exposure to rickettsioses (in all the, IFA-IgG titer was ≥ 1:128); SFG rickettsioses in 33/50 (66%), ST in 1/57 and for both SFG and ST in 14/57 (28%) (all 14 had higher titres for SFG) and none for *R. typhi*.

### Group two

During the four month period, all 49 patients (all males) who presented with acute febrile illness and admitted to the Palali Military hospital were recruited. Their mean age was 28.66 (SD: 7.5) years and duration of fever was 8.5 days (SD 3.2). Of the 49, 33 (67.3%) were serologically positive for acute rickettsioses (in all of them the IgG dilution titre was > 1:1024). 26/49 (53%) were due to ST and 7/49 (14%) were due to SFG rickettsioses. All responded well to doxycycline or doxycycline and azithromycin in combination. Among the study participants, 32/49 (65%) had 1:128 IFA-IgG titres for SFG rickettsioses. None of them were positive for *R. typhi*. Out of the 26 patients who had ST, 10 had been sleeping in a single field camp for two nights during field activity and all of them were admitted within a space of two weeks from the first patient. None of these patients had used insect repellents or doxycycline prophylaxis. Out of the patients with rickettsioses, 12/33(36%) had eschars, 20/33 had pneumonitis (Diagnosed by clinical and radiological features) and 6 of them had reduced capillary oxygen saturation (<93%) on air. 8 patients had myocarditis (Diagnosed by ECG changes and by ECHO cardiography) and one patient had meningo-encephalitis (Diagnosed by clinical features and EEG. This patient had an eschar suggesting the aetiological diagnosis of rickettsioses. Therefore lumbar puncture was not attempted). Two patients who had multiple organ involvement and needed intensive care management. Of the 16 non-rickettsial patients, 4 had vivax malaria, 6 had undetermined upper respiratory tract illness, 5 had leptospirosis and one had salmonella infection.

In the group one, 84% showed evidence of exposure to SFG rickettsioses (transmitted by hard ticks) compared to ST (transmitted by mites). These military personnel were originally from either wet or dry zones of low country in Sri Lanka. In the group two, 79% had high titres for ST and 21% had high titres for SFG rickettsioses. All of them had clinical response with anti-rickettsial antibiotics confirming the diagnosis of acute rickettsioses. The two military groups had been in uniforms during most of the time, they had been sleeping in scrub land and they had not been taking precautions against insect bites.

The outcome of group two suggested risk of the military mainly for ST outbreaks similar to that was observed during World Wars. As this study was carried out over a period of only 4 months and the sample consisted of several soldiers who were deployed in the same locality the predominant species that is responsible for acute rickettsioses in operational areas cannot be confirmed. However soldiers are known to run the risk of acquiring ST infection when they come in contact with ‘mite islands’.

We observed 84% sero-prevalence for rickettsioses in the group one. A 56.3% mean seroprevalence rate for rickettsioses has been documented in a previous community study conducted in four geographical regions of the country [5]. In that study, the IgG cutoff titre had been ≥1:64. However we observed ≥1:128 IgG titres among the first study group. The reasons for high titres against SFG rickettsioses in this group could not be assertained with the currently available diagnostic facilities. However, the study populations had no documented febrile illness during their service period. Therefore, one possibility for this observation could be repeated exposure to non-pathogenic rickettsial species resulting in development of nonspecific cross reacting antibody titres in them [7]. Similar observations were made among workers of a wild life conservation park of Sri Lanka, who had many tick bites almost daily for several years, and had almost 100% sero-prevelence for SFG rickettsioses, but had no evidence of febrile illness (unpublished data). If our assumption would be a likely possibility, it is important to study and identify such rickettsial agents that are harboured by wild ticks because some of the rickettsial organisms initially thought to be non-pathogenic have later being identified as pathogenic [8]. Rickettsial sero-prevalence among populations is known to be determined by their occupations, activities involved or climatic conditions. A 57.3% sero-prevalence for SFG rickettsioses has been documented among rubber estate workers in Slim River, Malaysia during wet season in December 1996 [9]. Screening of 169 US Army troops who participated in a 10 day training course in Botswana in 1999 has reported overall sero conversion rate of 14% (24/169) for SFG rickettsioses [10]. During the period the second study, the area had mostly a dry weather until mid September. There were two weeks of heavy rains during the latter part of September and in early October and the area remaind fairly wet for the next two months.

These soldiers had minimal understanding on rickettsial diseases and its mode of transmission. They had been in military uniforms and were sleeping in the scrub land as they were engaged in active militory efforts. They had not been carring any sleeping material with them during the field activities.

## Conclusion

Exposure to rickettsial disease is common among soldiers who are deployed in Nothern Sri Lanka. The high seroprevalence for SFG rickettsioses with no anticedent febrile illness warrent a closer look most appropriately by studying the offending ticks in order to assertain the rickettsial species that are carried by them. As exposure to rickettsial infections are high among the military, preventive measures such as use of insect repellents and doxycycline prophylaxis should be strongly recomended for them. The higher background IgG antibody titres against SFG rickettsiosdes is likely to interfere with serological diagnosis of acute SFG rickettsioses among these populations.
